# Determination and Comparison of Soybean Lecithin and Bovine Brain Plasmalogens Effects in Healthy Male Wistar Rats

**DOI:** 10.3390/ijms24087643

**Published:** 2023-04-21

**Authors:** Yuliya S. Sidorova, Varuzhan A. Sarkisyan, Nikita A. Petrov, Yuliya V. Frolova, Alla A. Kochetkova

**Affiliations:** Federal State Budgetary Scientific Institution “Federal Research Centre of Nutrition, Biotechnology and Food Safety”, Ustyinsky Proezd, 2/14, 109240 Moscow, Russiakochetkova@ion.ru (A.A.K.)

**Keywords:** phospholipids, ether lipids, cognitive functions, neuromuscular junction, brain lipids, hypolipidemic effect

## Abstract

The aim of this study was to investigate the effects of soybean lecithin and plasmalogens concentrating on a variety of physiological tests and biochemical analyses in healthy Wistar rats. For six weeks, male Wistar rats were given a standard diet that included plasmalogens or soybean lecithin. We measured anxiety levels, overall exploratory activity, short- and long-term memory, cognitive abilities, and grip strength. Lecithin increased significantly anxiety and enhanced memory and cognitive functions. Plasmalogens significantly improved appetite and increased grip strength. When compared to plasmalogens, lecithin significantly raised HDL levels while lowering LDL levels. The plasmalogens group showed a significant increase in the C16:0DMA/C16:0 ratio, which led us to assume that plasmalogen consumption could increase their synthesis in neural tissue. The study’s findings imply that, despite their various modes of action, soy lecithin and plasmalogens may both be significant nutritional components for enhancing cognitive functions.

## 1. Introduction

The widespread nature of neurodegenerative disorders is one of the world’s major challenges. Even in the early stages, the lipid profile of patients with neurodegenerative diseases undergoes significant changes [1]. In particular, the level of individual phospholipids as components of neural cell membranes varies significantly [2]. In the event of timely detection of the disease, diet therapy can have a positive impact on its development. In this regard, methods of nutritional correction of neurodegenerative disorders using phospholipids are the subject of ongoing studies [3].

One of the most promising classes of phospholipids is plasmalogens, unique phospholipids that contain an alk-1-enyl (vinyl ether) bond. Recent evidence suggests that the level of plasmalogens is highly correlated with the development of several neurological disorders such as Alzheimer’s disease, Parkinson’s disease, and Niemann-Pick type C [4]. 

Although meat and fish consumed in a diet contain substantial amounts of plasmalogens [5], a systemic understanding of their nutritional role for a generally healthy population is still lacking. Most studies in this field are only focused on their absorption and distribution capabilities, such as lymphatic absorption [5] and accumulation in erythrocytes [6]. Recent studies showed that oral administration of plasmalogens could improve cognition in Alzheimer’s disease mouse models [7,8]. Additionally, one preliminary clinical study showed the positive effects of plasmalogens on people with mild cognitive impairment [9]. The authors of these studies indicate a specific nutritional role of plasmalogens in the development of neurodegenerative disorders. 

On the other hand, some authors reported similar results for plant acyl-phospholipid mixtures containing no plasmalogens [3]. Of them, the most common and well-studied is soybean lecithin, which can improve memory, cognition, and motility. However, no studies were conducted to compare the biological activity of plasmalogens with that of soybean lecithin when consumed with food by a healthy general population or by healthy laboratory animals. It is therefore necessary to understand whether plasmalogens and acyl-glycerophospholipids have different nutritional roles. Thus, in this study, we investigated the differences between the effects of orally supplemented plasmalogens concentrate and soybean lecithin in a set of physiological tests and biochemical analyses in healthy Wistar rats.

## 2. Results

### 2.1. Body Weight and Food Intake 

Body weight gain increased gradually in all groups of animals during the experiment. The general condition of all animals in terms of appearance, haircoat quality, food, and water intake, behavior, and growth rate was inspected daily and found satisfactory throughout the experiment.

**Figure 1:**
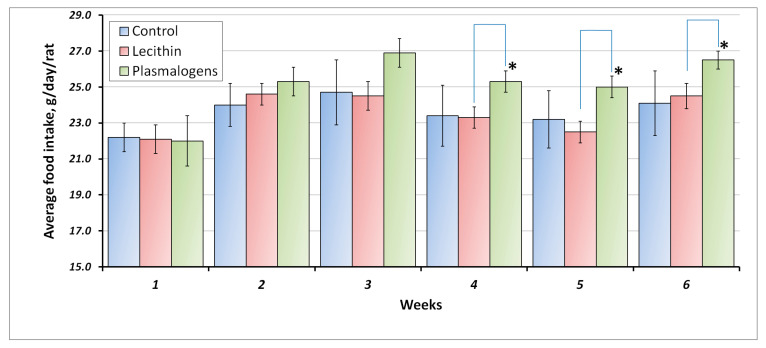
Daily food intake. Values are mean ± SEM of 15 rats per group * one-way ANOVA *p* < 0.05 versus Lecithin.

The average food intake (g/day/rat) of all groups at 1-week intervals is shown in Figure 1. 

The average consumption of phospholipids was 166 ± 1.8 mg/kg/day and 157 ± 2.2 mg/kg/day for the Plasmalogen and Lecithin groups accordingly. Values were obtained by calculations including the average body weight and the average food intake.

Starting from week 4 of the experiment, the food intake of the animals receiving plasmalogens was significantly higher than that of the animals receiving lecithin.

**Figure 2:**
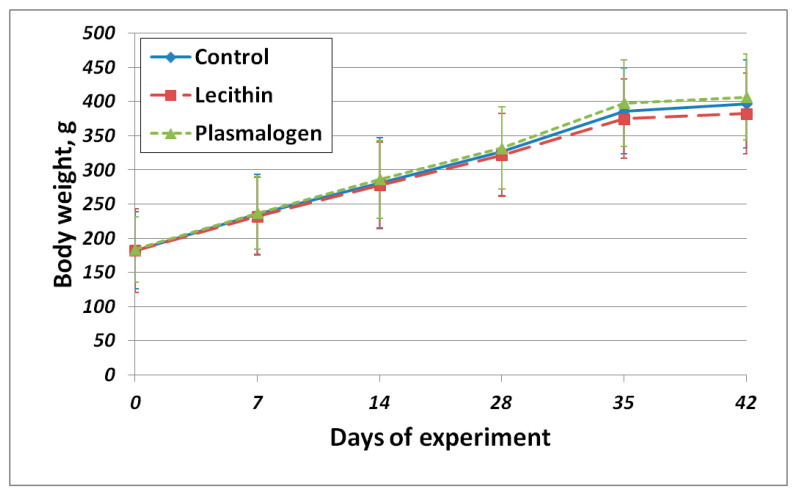
Average body weight gain. Values are mean ± SEM of 15 rats per group.

The body weight gain of all animals was normal for animals of this species and age (Figure S1). There were no significant differences in body weight gain among all groups. The body weight gain curve is shown in Figure 2. 

Despite greater food intake, the body weight gain of the Plasmalogen group animals did not differ significantly from other groups.

### 2.2. Physiological Tests

The results of the open field tests conducted at the beginning and on day 29 of the experiment and those of the elevated plus maze tests conducted at the beginning and on day 30 of the experiment are shown in Table 1.

After the first test, animals were randomly divided into groups with similar activity coefficients (AC) (*p* > 0.05), and animals with AC less than 1.0 were rejected (Tables S1 and S3). By day 29 of the experiment, the behavior of all animals changed, they moved mostly along the border of the box or spent all the time in one of the corners (Table S2). The AC obtained in test 1 differs significantly from that obtained in test 2. Moreover, AC in the lecithin group differs significantly from that in the control and plasmalogen groups. Thus, the animals receiving lecithin were the least active. As shown in Table 1, there were no significant differences in the time intervals spent by animals in the open and closed arms of the maze between the groups. On day 30 of the experiment, the behavior of all animals changed: they did not move much between arms, spending a long time in closed arms (Table S4). Also, the animals in the lecithin group spent significantly less time in open arms in comparison with Test 1, which may contribute to an increase in the anxiety level of the animals in this group. The inclusion of plasmalogens into the diet did not lead to a significant increase in anxiety level.

**Figure 3:**
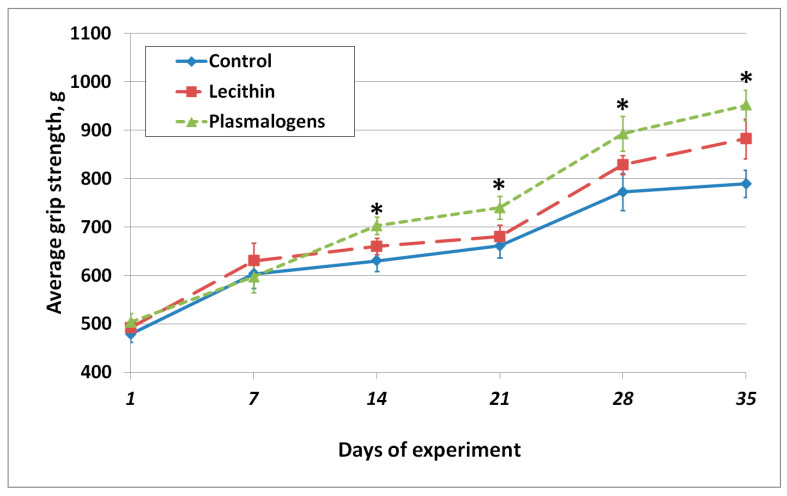
Dynamics of average grip strength values. Values are mean ± SEM of 15 rats per group * one-way ANOVA *p* < 0.05 versus Control.

The grip strength was monitored throughout the experiment. The results are shown in Figure 3.

As shown in Figure 3, starting from day 14 of the experiment, the grip strength of the Plasmalogen group animals was significantly higher than in the Control group. 

**Figure 4:**
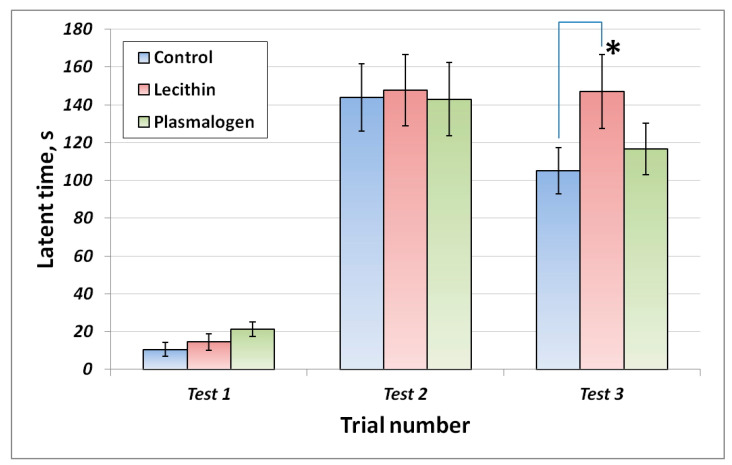
Passive avoidance test results. Values are mean ± SEM of 15 rats per group Test 1 was conducted on day 11 Test 2 is the short-term memory test conducted on day 12 Test 3 is the long-term memory test conducted on day 33 * one-way ANOVA *p* < 0.05 versus Control.

Short- and long-term memory were assessed in the passive avoidance test (Figure 4).

On the first day of testing, all animals entered the dark room (100% training success). On the second day, there were no significant differences in the latency time parameter between the groups. In the third test in 3 weeks, the latent time of the animals in the lecithin group was significantly higher than that in the control group. 

**Figure 5:**
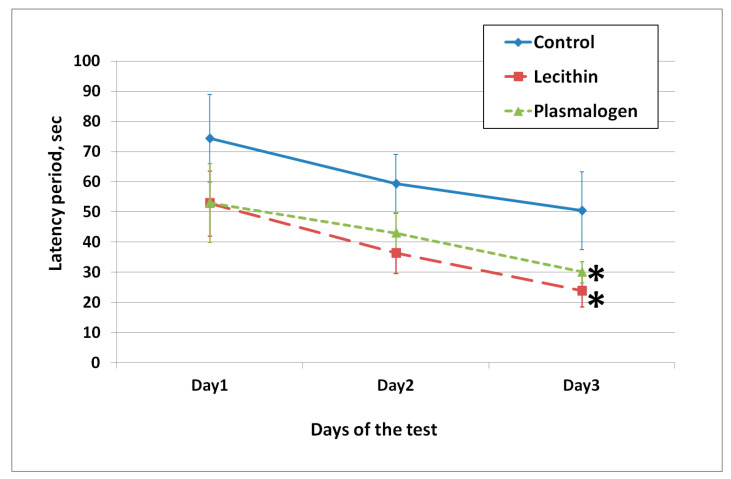
Average swimming time in Morris water maze. Values are mean ± SEM of 15 rats per group * one-way ANOVA *p* < 0.05 versus Control.

The cognitive functions of the animals were assessed in a “Morris water maze”. The training was conducted on days 37–39 of the experiment. The results are shown in Figure 5. In the first 2 days, there was no significant difference between the lecithin, plasmalogen, and control groups (*p* > 0.05).

On the third day, the latency period was shorter in the lecithin supplementation group than in the control group. The latent swim time to the platform of the lecithin group animals was significantly lower than that of the control group animals.

### 2.3. Biochemical Parameters

The data on lipid metabolism parameters, lipid hydroperoxides, and total antioxidant activity are shown in Table 2.

As shown by the data given, the lecithin group’s HDL value was significantly higher than that of the control group, while the LDL level was significantly lower. HDL mediates the function of reverse cholesterol transport and other protective functions, including the transport of polyunsaturated fatty acids, has antioxidant activity, and regulates the activity of glucocorticoids. Increased content of HDL in the blood serum of the animals receiving lecithin reflects certain beneficial effects on lipid metabolism. The total cholesterol and LDL levels of the Plasmalogen group animals were significantly lower in comparison with the Control group. A significant increase in lipid hydroperoxide levels and a decrease in total antioxidant activity were observed in the plasmalogen group in comparison with the control group. The animals from the lecithin group only exhibited a significant increase in lipid hydroperoxide levels in comparison with the control group. No significant differences were found between the plasmalogen and lecithin groups in any of the described biochemical parameters.

The differences in the composition of fatty acids and fatty aldehydes in rat brain tissue are provided in Table 3.

A post hoc analysis revealed that the lecithin and plasmalogen groups, when compared to the control group, had significantly lower levels of docosatetraenoic acid (C22:4) and lignoceric acid (C24:0), but higher levels of docosahexaenoic acid (C22:6 ω-3) and ω-3 to ω-6 ratios from total and very long chain fatty acids (VLCFA). In addition, the lecithin and plasmalogen groups had significantly higher levels of dihomo-gamma-linolenic acid (C20:3 ω-6) and docosapentaenoic acid (C22:5 ω-6) compared to the control group. As can be seen from Table 3, the only distinctive characteristic of the plasmalogen group was a higher level of C18:1 (U = 1, Z = −3.44, *p* < 0.001) and a lower level of C20:4 (U = 11, Z = 2.56, *p* = 0.008) compared to the control group. The most remarkable aspect of these data is the increase in the C16:0DMA/C16:0 ratio (U = 12, Z = −2.65, *p* = 0.006) in the plasmalogen group compared to the lecithin group.

## 3. Discussion

In the present study, we examined differences between the nutritional roles of plasmalogens and soybean lecithin in a healthy Wistar rat model. In general, our findings suggest that soybean lecithin and plasmalogens, when consumed as dietary components, have diverse modes of action.

The most significant physiological outcomes of soybean lecithin consumption were enhancements of memory and cognitive functions in rats, along with a decrease in activity and an increase in anxiety. Meanwhile, plasmalogen consumption enhances the appetite and increases nonspecific endurance. There are two possible explanations for these findings. Higher food intake might be caused by less energy accessibility since the sn-1 vinyl bond of plasmalogens stays intact after oral administration [10,11]. On the other hand, plasmalogens are a source of endocannabinoids, particularly anandamide [12], which are known to increase appetite and enhance endurance [13,14,15].

The combination of both behavioral tests shows an increase in the anxiety levels of the animals receiving lecithin. Administration of plasmalogens did not impair exploratory activity or anxiety levels, which may support the beneficial effects of plasmalogens on these physiological parameters in comparison with soybean lecithin.

Soybean lecithin treatment has shown a significant increase in the HDL level and a decrease in the LDL level, whereas treatment with plasmalogens led to a significant decrease in LDL only. Moreover, in these groups, the markers of oxidative processes were significantly higher than in the control group.

We also demonstrated differences in the changes of fatty acids and fatty aldehydes in brain tissue following the consumption of these phospholipids. For the first time, it was shown that the concentration of plasmalogens in rat brains (due to C16:0 fatty aldehyde concentration) may be increased by the consumption of plasmalogens.

The main advantage of this study is that the nutritional role of phospholipids was investigated as part of a food product with an optimal fatty acid profile. Moreover, two types of phospholipids were compared under the same conditions using rats that had been preliminary divided into groups using the “open field” and “elevated plus maze” tests. Animals’ behavior in the “open field” test is determined by the relationship between defensive and exploratory tendencies. The “elevated plus maze” test is used to study animals’ behavior under variable stress conditions, i.e., with the free choice of comfortable conditions, and thus allows us to evaluate their anxiety level. The evaluation of behavior is based on a conflict between the animal’s desire to explore and its fear of open spaces. The combination of these tests precludes unexpected biases caused by differences in the intensity of fear, anxiety reactions, and total exploratory activity of animals at baseline.

These results are consistent with the findings of other studies, where the administration of soybean lecithin or its fraction had a positive effect on memory and cognitive function in rats but a negative one on their activity and anxiety [16,17]. Furthermore, our results are in line with a recent study [18] indicating an association between plasmalogens and the function of the neuromuscular junction. However, in contrast to papers published since the study was designed and carried out [7,8], no evidence of a significant positive effect on memory and cognitive function in rats was found. A possible explanation for this could be that plasmalogen metabolism and antioxidative status in the setting of Alzheimer’s disease differ from those in a healthy state [2].

The causative factor of a positive effect on the cholesterol level in this study cannot be accurately determined because several factors might have contributed to this result, such as ω-3 fatty acids, astaxanthin, phospholipids, physiological tests themselves, or a combination of these factors.

Higher levels of lipid hydroperoxides as well as lower antioxidant activity in the blood of the Lecithin and Plasmalogen groups of animals compared to the Control group may be explained by the high DHA content in their diets [19]. It is therefore possible that astaxanthin is inefficient as an antioxidant in this system. Thus, additional vitamin E is needed to neutralize the negative effects of a diet high in ω-3 fatty acids.

Changes in the fatty acid composition of the brain are consistent with the data on the ability of fatty acids in the composition of triglycerides or phospholipids to pass through the blood-brain barrier and accumulate in the brain [20]. A high C16:0DMA/C16:0 ratio was observed in the group receiving plasmalogens after overnight fasting. Considering this, high turnover rates and short half-life times of plasmalogens in rat brains [21], it might be hypothesized that plasmalogen consumption can elevate their synthesis in neural tissue.

## 4. Materials and Methods

### 4.1. Samples

In our study, we used two samples of functional food products (Table 4) represented by product 1 (lecithin group) or product 2 (plasmalogen group) containing 40% of the lipid phase. The lipid phase consisted of a mixture of high-oleic sunflower oil (88.8% *w*/*w*), coconut oil (6.3% *w*/*w*), and microalgae oil (4.9% *w*/*w*, Life’s Omega^TM^ 60) to provide the presence of 9.0% ω-6 fatty acids, 2.0% of ω-3 fatty acids (from which, 90% from docosahexaenoic acid). These products are also characterized by high content (70% *w*/*w*) of oleic acid and medium-chain fatty acids. The combination of oils for the lipid phase was chosen to meet the requirements of the FAO expert consultation report (Joint, FAO (2010). Fats and fatty acids in human nutrition. Report of an expert consultation, 10–14 November 2008, Geneva).

Pectin, carboxymethyl cellulose, and locust bean gum were used in both products as structuring agents. Astaxanthin (DSM, Heerlen, Netherlands) was added to the composition of both products to prevent the oxidation of unsaturated fatty acids.

The products differed by the type of phospholipids used. Product 1 contained commercial soybean lecithin (de-oiled, powdered soybean lecithin containing a mixture of phospho- and glycol-lipids; acetone insoluble substance content > 96%; LECIGRAN 1000p Cargill, Düsseldorf, Germany); product 2 contained plasmalogens concentrate in an amount of 0.1% of the diet, according to [6]. Bovine brain white matter was used as a plasmalogen-rich source. Phospholipids were extracted by the hexane:isopropanol method described in [22], followed by precipitation of the total lipid fraction in cold acetone (4 °C). Plasmalogens were concentrated with the phosphatidylethanolamine fraction in aqueous ethanol as described by Wu and Wang [23] with the following modifications: acetone precipitates were dried under vacuum, resuspended in ethanol (1:10 *w*/*v*), and dissolved by sonication (30–60 s). Sonicated samples were stored in a cold bath (2–4 °C) for 1 h and centrifuged (8000× *g*, 15 min, 4 °C). Supernatants were collected and cooled to −27 °C for 2 h to form a lipid precipitate (the plasmalogen-containing fraction). The precipitate was centrifuged (950× *g*, 15 min, 4 °C) and collected. The phospholipid compositions of fatty acids and fatty aldehydes (from plasmalogens) measured by gas chromatography are shown in Table 3.

A dry mixture of pectin, carboxymethylcellulose, and locust bean gum was added to a container with water at a temperature of 45 °C at concentrations of 0.25 g/100 g, 0.25 g/100 g, and 0.4 g/100 g, respectively. Under stirring at a speed of 500 rpm on a magnetic stirrer, the water temperature was set to the value required for the complete hydration of dry substances (75–80 °C). After that, the mixture was cooled to 50 °C, and with vigorous stirring (Heidolph SilentCrusher M homogenizer (Heidolph Instruments GmbH & Co., Schwabach, Germany), 16,000 rpm, 5 min), the lipid phase (containing either lecithin or plasmalogen) was slowly added until the formation of a coarse emulsion. The coarse emulsion was then processed in a universal Stephan UMC5 laboratory machine (pressure 0.5 bBar, 3000 rpm) for 15 min during cooling from 35 °C to 15 °C until a stable emulsion was formed. The samples were stored at +4 °C in closed containers until they were added to the feed of the laboratory animals.

### 4.2. Animals

Fifty-five 4-week-old male Wistar rats with a body weight of 80 ± 5 g were purchased from the Stolbovaya Nursery of laboratory animals of the Scientific Centre for Biomedical Technology of the Federal Medical and Biological Agency.

The animals were housed two per cage in polycarbonate cages and kept under a controlled 12-h light/dark cycle. Room temperature and relative humidity were maintained at 23 ± 2 °C and 60% ± 5%, respectively.

The experimental approach based on the preliminary division of the animals according to their performance in the “Open field” (OF) and “Elevated plus maze” (EPM) tests was used in this study. The statistical validity and verifiability of the results may be increased by using a combination of different behavioral tests. After one week of acclimation, all rats were subjected to the “Open field” (OF) and “Elevated plus maze” (EPM) tests to preliminarily divide the animals according to their adaptation to stress ability.

### 4.3. Open Field Test

The test was performed before the start and on day 29 of the experiment. In the OF test, an animal is placed in an unknown open field. The open field box was a black square arena (90 cm × 90 cm) surrounded by gray walls (40 cm) and divided into one central zone and two peripheral zones.

The animals were placed in the corner and allowed to explore the box for 3 min. To evaluate the behavior, the following parameters were used: horizontal (number of transitions between zones) and vertical (number of rearings) activity, the latency of the first move, and the latency of the first entry into the central zone. The test was conducted during the period of the animals’ minimal activity (between 10 a.m. and 3 p.m.). The activity coefficient (AC) was calculated according to [24] as:

$$\mathrm{AC}=\frac{\mathrm{ZT}+R}{L_{\mathrm{fm}}+L_{c}}$$

where ZT is the total number of zone transitions, R is the total number of rearings, L_fm_ is the latency of the first move, L_c_ is the latency of the first entry into the center.

### 4.4. Elevated plus Maze

The test was performed on the day following the open-field test and on day 30 to evaluate changes in the animals’ anxiety levels. The elevated plus maze consisted of two opposite arms (45 cm × 10 cm) crossed with two opposite enclosed arms of the same size with 50 cm high walls. The maze was elevated 65 cm above the floor in a dimly lit room.

The rats were placed in the center of the maze facing the closed arm and were allowed to explore the maze for 5 min. The number of entries and the time spent in each arm of the maze were recorded. The data were expressed as a percentage of time spent in the open arms, constituting the index of anxiety, and the total number of closed-arm entries, constituting the index of total activity [25]. 

Movements of the animals in the OF and EPM tests were registered using the Smart v3.0.04 software (Panlab Harvard Apparatus, Barcelona, Spain). 

### 4.5. Design of the Experiment

After preliminary tests, animals with AC less than 1.0 were rejected (10 animals were rejected). Remaining 45 rats were randomly divided into three experimental groups (n = 15 animals per group) and received different experimental diets for 6 weeks: the control group was fed a standard diet; Lecithin group was fed a diet containing 130 g Product1/kg and Plasmalogen group was fed a diet containing 130 g Product2/kg (Table 5).

The animals were provided with food and water ad libitum. The body weight was measured weekly; food intake was measured three times a week.

### 4.6. Grip Strength Test

The animals’ grip strength was measured weekly. Their neuromotorics (muscle tone) were assessed by determining the forelimb grip strength of the rats. The test also allowed us to assess both decreases and increases in the static component of nonspecific endurance.

The test was performed with a grip strength meter (Bioseb, Vitrolles, France). Each animal was held by the body and brought near the bar letting them grab it with both forepaws and then gently pulled back until they released the bar. The rats were tested three times in a row; the results of the three tests were averaged for each rat. The resting period between each attempt was 1 min. The unit of force used was gram-force.

### 4.7. Passive Avoidance Test

A passive avoidance test was used to assess the behavior, and short-term and long-term memory of the animals. The apparatus consisted of a large white illuminated compartment and a small black dark compartment separated by a door.

On day 10 of the experiment in the training session, the animals were placed in the light compartment and explored the apparatus for five minutes. On the following day (day 11), when the animals entered the dark compartment, the door was closed and they received an electric shock (0.4 mA, 4 s). The latent period of staying in the light compartment was recorded. In the retention phase, 24 h later (short-term memory) and in 3 weeks (long-term memory), the animals were placed in the light compartment, and the latency of entering the dark compartment was recorded.

### 4.8. Water Morris Maze

The cognitive functions of the animals were evaluated in the Morris water maze on the 37th–39th days of the experiment. The test was conducted in a round pool 120 cm in diameter with walls 60 cm high. The water temperature was maintained at the level of 26 ± 2 °C. The pool was divided into 4 quadrants and a round refuge platform was placed in the pool 1 cm below the water in one of the quadrants and the position was fixed. The rat was placed in the sector opposite to that the platform. The animal was allowed to explore the pool freely for 180 s. If it didn’t find the platform, it was carefully guided to it and kept on the platform for 20 s. The next round of training was initiated in 180 s, and the score of this round was 180 s. All rats were trained 3 times/day for 3 consecutive days. The average time that the rat needed to find the platform was taken as the search latency of the day [26].

### 4.9. Biochemical Analyses

At the end of the experimental period, the animals were sacrificed by CO_2_ asphyxiation after an overnight fast. Blood samples were taken from the inferior vena cava, collected into microfuge tubes, and allowed to clot. The samples were centrifuged at 500× *g* for 15 min; serum was separated, aliquoted, and stored at –20 °C for the relevant assays.

Total cholesterol (TC), triglycerides, and high-density lipoprotein (HDL) levels in the serum of all animals were measured using an automatic biochemistry analyzer (Konelab 20i, Thermo Scientific, Waltham, MA, USA) under the standard procedure. The low-density lipoprotein (LDL) concentration was calculated as follows [27]:

$$\mathrm{LDL}=\frac{3}{4}\left(\mathrm{TC}-\mathrm{HDL}\right)$$

The state of lipid peroxidation processes in the animals was assessed by the content of lipid hydroperoxides in blood serum. The method is based on the reduction of the peroxide group followed by colorimetric measurement. Fe^2+^ was used as a reduction agent. 100 µL of blood plasma was mixed with 900 µL of FOX reagent (100 µM xylenol orange, 25 mM sulfuric acid, 250 µM ammonium ferrous sulfate) and incubated at room temperature for 30 min with constant agitation. The lipid hydroperoxides content was measured at 560 nm using an H_2_O_2_ standard curve.

Total antioxidant activity was measured by the spectrophotometric method: an analysis of the reaction product, a complex of ferrous ion and 2,4,6-tris(2-pyridil)-s-triazine (Fe^2+^-TPTZ), was performed. 62 µL of blood serum was added to 1.86 mL of FRAP and absorption was measured at wavelength 593 nm for 4 min.

### 4.10. Brain Tissue Collection and Preparation

Immediately after blood collection, the brain was removed and placed on a cold metal plate. Hemispheres were dissected, rinsed with normal saline, weighed, then homogenized in 10 mL of a cold mixture of chloroform/methanol (2:1) with 0.05% butylated hydroxyanisole as an antioxidant.

### 4.11. Fatty Acid and Fatty Aldehyde Analysis

Lipids were extracted from the rat brain preparations by the Folch method [28] and analyzed by gas chromatography [29] with an Agilent 7890A Gas Chromatograph with a flame-ionization detector. The column was Agilent J&W GC Select FAME (50 m × 25 mm × 0.25 μm). The temperatures of the detector and the injector were maintained constant at 260 °C and 240 °C, respectively, and the oven temperature was increased from 140 (maintained for 5 min) to 220 °C at 4 °C/min and maintained at 220 °C for 25 min. Nitrogen was used as the carrier gas at the flow rate of 0.9 mL/min.

### 4.12. Statistical Analysis

All analyses were carried out using SPSS 20. One-way ANOVA and Tukey’s post hoc tests were used for normally distributed data and Kruskal–Wallis and Mann–Whitney tests were used for non-normally distributed data. *p* values < 0.05 were considered significant.

## 5. Conclusions

This study aimed to examine the mode of action of phospholipids in healthy rats; therefore our findings cannot be extrapolated to different disease states. Nevertheless, this limitation may be a subject of further research.

Overall, this study supports the idea that both soybean lecithin and plasmalogens may be important nutritional factors for enhancing cognitive functions, despite their different modes of action. In addition, the results of our study indicate the possibility of clinical use of plasmalogens in the treatment of disorders caused by altered neuromuscular junctions.

## Figures and Tables

**Table 1:** Open field test and elevated plus maze test results.

Group	Open Field		Elevated plus Maze			
	Activity Coefficient		Time in Open Arms, %		Total Number of Closed Arms Entries	
	Test 1	Test 2	Test 1	Test 2	Test 1	Test 2
**Control**	9.0 ± 1.2	4.3 ± 2.1 *	9.2 ± 2.9	7.2 ± 2.4	7 ± 1	6 ± 3
**Lecithin**	9.1 ± 2.6	1.6 ± 0.5 *^,#,∆^	10.0 ± 1.4	4.9 ± 1.9 *	7 ± 1	6 ± 3
**Plasmalogen**	8.8 ± 1.4	4.8 ± 1.5 *	11.5 ± 2.3	5.7 ± 2.2	6 ± 1	6 ± 3

Values are mean ± SEM of 15 rats per group; Test 1—before feeding with experimental diets; Test 2—on days 29 and 30 days of the experiment, respectively; one-way ANOVA * *p* < 0.05 versus Test 1; ^#^ *p* < 0.05 versus Control; ^∆^ *p* < 0.05 versus Plasmalogen.

**Table 2:** Biochemical parameters.

Parameter	Groups		
	Control	Lecithin	Plasmalogen
**Cholesterol, mmol/L**	2.02 ± 0.07	1.87 ± 0.1	1.73 ± 0.1 *
**HDL, mmol/L**	0.58 ± 0.020	0.66 ± 0.03 *	0.62 ± 0.04
**LDL, mmol/L**	1.07 ± 0.04	0.92 ± 0.05 *	0.83 ± 0.05 *
**Triglycerides, mmol/L**	1.39 ± 0.17	1.48 ± 0.18	1.39 ± 0.14
**Lipid hydroperoxides, μmol** **/mL**	0.19 ± 0.01	0.25 ± 0.02 *	0.24 ± 0.01 *
**Total antioxidant activity, mM**	1.03 ± 0.03	0.97 ± 0.05	0.88 ± 0.05 *

Values are mean ± SEM of 15 rats per group, * one-way ANOVA *p* < 0.05 versus Control.

**Table 3:** Main fatty acids and fatty aldehydes composition of rat brain.

Index	Group		
	Control (n = 8)	Lecithin (n = 9)	Plasmalogen (n = 9)
**C18:1**	18.608 ± 0.750	19,501 ± 19.501	20,305 ± 0.530
**C20:3**	0.394 ± 0.045	0.518 ± 0.116 ^a^	0.502 ± 0.058 ^a^
**C20:4**	10.080 ± 0.460	9.629 ± 0.496	9.517 ± 0.297 ^a^
**C20:5**	0.099 ± 0.053	0.041 ± 0.026	0.057 ± 0.051
**C22:4**	3.795 ± 0.614	1.504 ± 0.896 ^a^	1.192 ± 0.193 ^a^
**C22:5**	0.133 ± 0.034	0.235 ± 0.075 ^a^	0.227 ± 0.065 ^a^
**C22:6**	9.307 ± 0.328	12.373 ± 1.181 ^a^	12.698 ± 0.413 ^a^
**C24:0**	3.923 ± 0.189	3.289 ± 0.289 ^a^	3.241 ± 0.093 ^a^
**C16:0DMA/C16:0**	0.156 ± 0.013	0.160 ± 0.008	0.173 ± 0.017 ^b^
**VLCFA ω3/ω6**	0.89 ± 0.05	1.24 ± 0.14 ^a^	1.26 ± 0.05 ^a^
**Total ω3/ω6**	0.87 ± 0.05	1.21 ± 0.13 ^a^	1.21 ± 0.05 ^a^

VLCFA—very long chain fatty acids, DMA—dimethyl acetal, ^a b^ Mann-Whitney two-sided *p* < 0.05 between groups Control, Lecithin, and Plasmalogen, respectively.

**Table 4:** Composition of the developed functional foods.

Components	Content, g/100 g	
	Product 1	Product 2
Lipid phase, including	40	40
Oil mixture	37.87	37.87
Astaxanthin	0.03	0.03
Soybean lecithin	2.1	-
Plasmalogenes concentrate	-	2.1
Water phase. including	60	60
Distilled water	59.1	59.1
Pectin	0.25	0.25
Carboxymethyl cellulose	0.25	0.25
Carob bean gum	0.4	0.4

**Table 5:** Distribution of animals by groups.

Parameter	Groups		
	Control	Lecithin	Plasmalogen
**Body weight, g**	182 ± 3	182 ± 2	183 ± 4
**Activity coefficient**	9.03 ± 1.2	9.11 ± 2.6	8.8 ± 1.4
**Time in open arms, %**	9.2 ± 2.9	10.0 ± 1.4	11.5 ± 2.3
**Total number of closed arm entries**	7 ± 1	7 ± 1	6 ± 1

Values are mean ± SEM of 15 rats per group.

## Data Availability

Data are however available from the authors upon reasonable request.

## Supplementary Materials

The supporting information can be downloaded at: https://www.mdpi.com/article/10.3390/ijms24087643/s1.
