# Supplementary material for: Determination and Comparison of Soybean Lecithin and Bovine Brain Plasmalogens Effects in Healthy Male Wistar Rats
Source: Int J Mol Sci. 2023 Apr 21;24(8):7643. doi: 10.3390/ijms24087643 (PMC10145545; doi:10.3390/ijms24087643)
Supplement: Supplementary file 1 [file ijms-24-07643-s001.zip › ijms-2305779-supplementary.pdf]

Supplementary materials to

Article

Determination and Comparison of Soybean Lecithin and Bovine Brain Plasmalogens Effects in Healthy Male Wistar Rats

Yuliya S. Sidorova[1,*], Varuzhan A. Sarkisyan[1], Nikita A. Petrov[1], Yuliya V. Frolova[1], Alla A. Kochetkova[1]

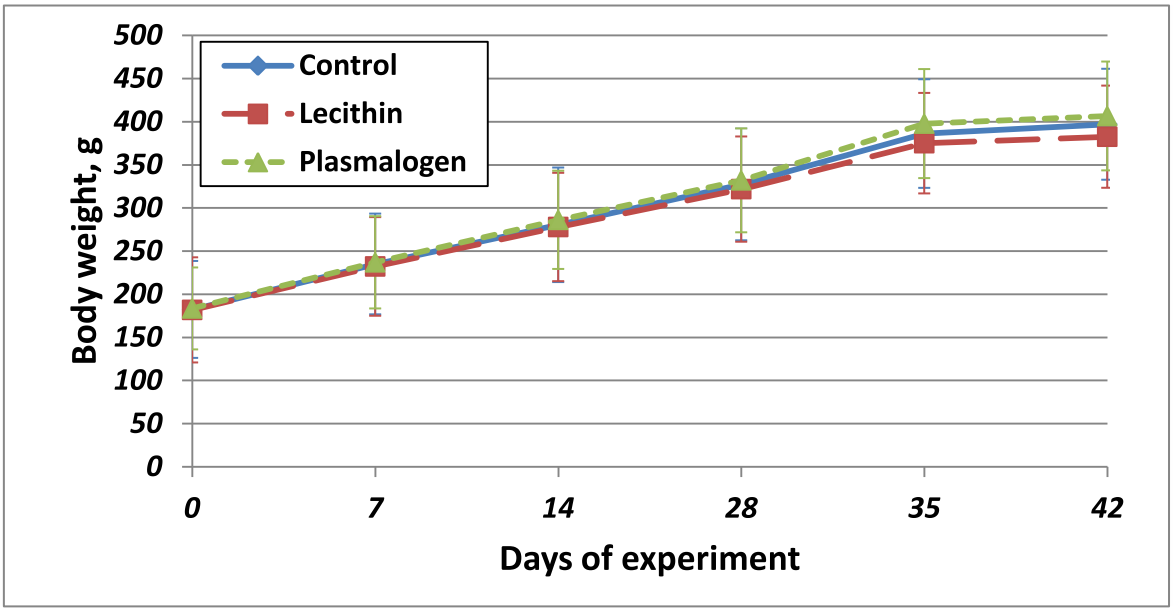

Figure S1. Dynamics of body weight, g. Values are mean ± SEM of 16 rats per group

Table S1. Open field test results (before the start of the experiment)

Group	Open field (before the start of the experiment)					
	Latency of the first move, sec	Latency 1st Entrance to Periphery, sec	Latency of the first entry into the central zone, sec	Number of rearings	Number of transitions between zones	The activity coefficient
Control	2.8±0.6	52±11	15±8	8.3±0.6	8.9±2.1	9.2±2.2
Lecithin	2.3±0.3	47±9	20±9	9.0±1.4	8.2±1.3	8.5±1.3
Plasmalogen	2.2±0.3	46±8	21±8	8.8±0.8	8.3±1.3	8.6±1.3

Table S2. Open field test results (29th day of the experiment)

Group	Open field (29th day of the experiment)					
	Latency of the first move, sec	Latency 1st Entrance to Periphery, sec	Latency of the first entry into the central zone, sec	Number of rearings	Number of transitions between zones	The activity coefficient
Control	8.8±3.2	118±23	171±6	4.2±1.3	3.5±2.0	4.3±2.1
Lecithin	51.2±20.7*,#	135±17	172±8	5.2±1.5	1.0±0.4#	1.6±0.5*,#
Plasmalogen	8.9±2.9	129±18	156±14	5.2±1.3	4.5±1.5	4.8±1.5

*P < 0.05 versus Control; # P < 0.05 versus Plasmalogen

Table S3. Elevated plus maze test results (before the start of the experiment)

Group	Elevated plus maze test (before the start of the experiment)				
	Time in Center, sec	Time in Open Arms, sec	Time in Closed Arms, sec	Total Distance, cm	Number of transitions between zones
Control	25.1±3.9	30.8±8.3	240±13	1258±80	19±3
Lecithin	24.8±4.4	30.1±4.6	245±7	1394±70	21±2
Plasmalogen	23.1±3.5	34.5±6.5	242±9	1320±70	19±2

Table S4. Elevated plus maze test results (30th day of the experiment)

Group	Elevated plus maze test (30th day of the experiment)				
	Time in Center, sec	Time in Open Arms, sec	Time in Closed Arms, sec	Total Distance, cm	Number of transitions between zones
Control	29.6±8.2	21.7±6.0	249±69	737±204	14±4
Lecithin	19.6±5.2	14.6±3.9	266±71	696±186	14±4
Plasmalogen	38.4±10.7	17.2±4.8	244±29	725±201	15±4
